# A new species of *Hyorrhynchus* Blandford, 1894 (Coleoptera, Curculionidae, Scolytinae, Hyorrhynchini) and two new records of xyleborine ambrosia beetles from Japan

**DOI:** 10.3897/zookeys.1284.195890

**Published:** 2026-07-14

**Authors:** Yogo Setiawan, Sarah M. Smith, Yositaka Sakamaki, Makoto Tokuda, Hagus Tarno, Kunihiko Hata

**Affiliations:** 1 The United Graduate School of Agricultural Sciences, Kagoshima University, 1–21–24 Korimoto, Kagoshima, 890–0065, Japan Department of Plant Pests and Diseases, Faculty of Bio-industry, Agriculture and Forestry, Universitas Brawijaya Malang Indonesia; 2 Department of Plant Pests and Diseases, Faculty of Bio-industry, Agriculture and Forestry, Universitas Brawijaya, Jl. Veteran, Malang, 65145, East Java, Indonesia School of Forest, Fisheries, and Geomatics Sciences, University of Florida Gainesville United States of America; 3 Department of Entomology, Michigan State University, 288 Farm Lane, 243 Natural Science Bldg., East Lansing, MI 48824, USA The United Graduate School of Agricultural Sciences, Kagoshima University Kagoshima Japan; 4 School of Forest, Fisheries, and Geomatics Sciences, University of Florida, 136 Newins-Ziegler Hall PO Box 110410, Gainesville, FL 32611, USA Entomological Laboratory, Faculty of Agriculture, Kagoshima University Kagoshima Japan; 5 Entomological Laboratory, Faculty of Agriculture, Kagoshima University, 1-21-24 Korimoto, Kagoshima, 890-0065, Japan Laboratory of Forest Protection, Faculty of Agriculture, Kagoshima University Kagoshima Japan; 6 Laboratory of Systems Ecology, Faculty of Agriculture, Saga University, 1 Honjo-Machi, Saga, 840-8502, Japan Laboratory of Systems Ecology, Faculty of Agriculture, Saga University Saga Japan; 7 Laboratory of Forest Protection, Faculty of Agriculture, Kagoshima University, 1-21-24 Korimoto, Kagoshima, 890-0065, Japan Department of Entomology, Michigan State University East Lansing United States of America

**Keywords:** Ambrosia beetles, Hyorrhynchini, *
Hyorrhynchus
*, Japan, key, new species, Scolytinae, taxonomy

## Abstract

*Hyorrhynchus takakumaensis* Setiawan, Hata & Smith, **sp. nov**. is described from Japan, and *Anisandrus auratipilus* Smith, Beaver & Cognato, 2020 and *Debus shoreae* (Stebbing, 1907) are reported from Japan for the first time. With these findings, the diversity of the Japanese Scolytinae fauna increases to three *Hyorrhynchus* species, four *Anisandrus* species, and three *Debus* species. A key to the Japanese *Hyorrhynchus* species is also provided.

## Introduction

Hyorrhynchini (Coleoptera: Curculionidae: Scolytinae) includes three genera, *Hyorrhynchus* Blandford, 1894; *Sueus* Murayama, 1951; and *Pseudohyorrhynchus* Murayama, 1950, all of which were erected to accommodate Japanese species. *Hyorrhynchus* is easily recognized by its divided eyes, with the upper portion located on the anterior face of the frons, seven-segmented antennal funicle, and by an elongate strongly compressed antennal club with two sutures (Beaver and Gebhardt 2005). *Hyorrhynchus* was established as a monotypic genus for *Hyorrhynchus lewisi* Blandford, 1894 from Japan (Blandford 1894). At present, *Hyorrhynchus* comprises ten valid species, all primarily distributed across Asia (Bright 2014; Johnson et al. 2026). These include *H. birmanus* Eggers, 1939, *H. drescheri* Eggers, 1936, *H. ebianensis* Huang & Yin, 1983, *H. elongatus* Eggers, 1939, *H. kalimpongensis* Maiti & Saha, 1989, *H. lewisi* Blandford, 1894, *H. sensarmai* Maiti & Saha, 1989, *H. shiva* Maiti & Saha, 1989, *H. tuberopectus* Huang & Yin, 1983, and *H. unicornis* Nobuchi, 1966 (Johnson et al. 2026). Among these species, *H. lewisi* and *H. unicornis* were originally described from Japan and remain the only species recorded in the country to date (Blandford 1894; Nobuchi 1966).

Scolytinae currently includes 30 tribes, 270 genera, and 6516 valid species worldwide (Johnson et al. 2026). The taxonomic history of Japanese bark and ambrosia beetles was summarized by Smith et al. (2018) and the most recent checklist lists 302 Scolytinae species (Goto 2009). Smith et al. (2018) recently reviewed the Japanese xyleborine fauna and later described two new species (Smith et al. 2022). However, an updated checklist of the beetle fauna has not been published since 2009. In this paper, we describe a new species, *Hyorrhynchus takakumaensis* Setiawan, Hata & Smith, sp. nov., and record the occurrence of two xyleborine ambrosia beetle species from Japan. We also provide a key to Japanese *Hyorrhynchus* species.

## Materials and methods

Specimens were collected using flight interception traps set in the Takakuma Experimental Forest of Kagoshima University, Tarumizu, Kagoshima Prefecture, located on the Osumi Peninsula, Kyushu Island, Japan. The new species was compared with all previously described *Hyorrhynchus* species based on photographs of type specimens (NHMUK, NHRS, NMNH) taken by SMS or through examination of the original descriptions. The holotype of *H. unicornis* (IPPN) was examined by YSN and SMS. *Hyorrhynchus tuberopectus* and *H. ebianensis* were compared based on the original descriptions and morphological illustrations, as images were unavailable. Two newly recorded species were identified by YSN, compared with photographs and diagnoses in Smith et al. (2020), and were subsequently confirmed and examined by SMS. Photographs were taken with a Nikon Digital Sight 1000 camera (Nikon Corporation, Yokohama, Japan). The photos were then combined with Zerene Stacker version 1.04 (Zerene Systems LLC, Washington State, USA). Measurements were taken using a Nikon stereomicroscope (SMZ1270) (Nikon Solutions Co., Ltd., Tokyo, Japan). All photos were improved with GIMP 3 software. Length was measured from pronotal apex to the apex of the declivity, and width was measured at the widest part of the specimen. The holotype and newly recorded specimens are deposited in the Entomological Laboratory, Faculty of Agriculture, Kagoshima University, Kagoshima, Japan.

### Abbreviations

**ELKU** Entomological Laboratory, Faculty of Agriculture, Kagoshima University, Kagoshima, Japan;

**IPPN** Institute for Plant Protection, National Agriculture and Food Research Organization (NARO), Tsukuba, Japan;

**NHMUK** Natural History Museum, London, UK;

**NHRS** Naturhistoriska riksmuseet, Stockholm, Sweden;

**NMNH** National Museum of Natural History, Smithsonian Institution, Washington, D.C., USA.

## Taxonomy

### Tribe Hyorrhynchini Hopkins, 1915

#### Genus Hyorrhynchus

Taxon classificationAnimaliaColeopteraCurculionidae

Blandford, 1894

7F4D1C8C-FAB8-5A49-A2F3-773AD4307118

##### Type species.

*Hyorrhynchus lewisi* Blandford, 1894.

##### Diagnosis.

Antennal club elongate and strongly compressed with two sutures; eyes divided, upper divisions of the eyes rounded triangular, situated on either side of front, lower divisions of the eyes hidden from above. Pronotum narrowed in front, widest at base, with sides rounded, more strongly rounded anteriorly, densely granulate above. Elytra wider than pronotum, and more than twice as long, dilated behind middle, separately rounded at base. In male, the frons is strongly longitudinally furrowed and expanded laterally at the apex into prominent angles; in female, the frons is slightly convex.

#### Hyorrhynchus takakumaensis

Taxon classificationAnimaliaColeopteraCurculionidae

Setiawan, Hata & Smith sp. nov.

BE27FB9A-4054-5EBB-88AB-29D9C4C595FB

https://zoobank.org/344496DB-D03B-4BE7-B564-F8C696399A73

Fig. 1A–F

##### Japanese name.

タカクマオオキクイムシ (Takakuma o-kikuimushi).

**Figure 1.**
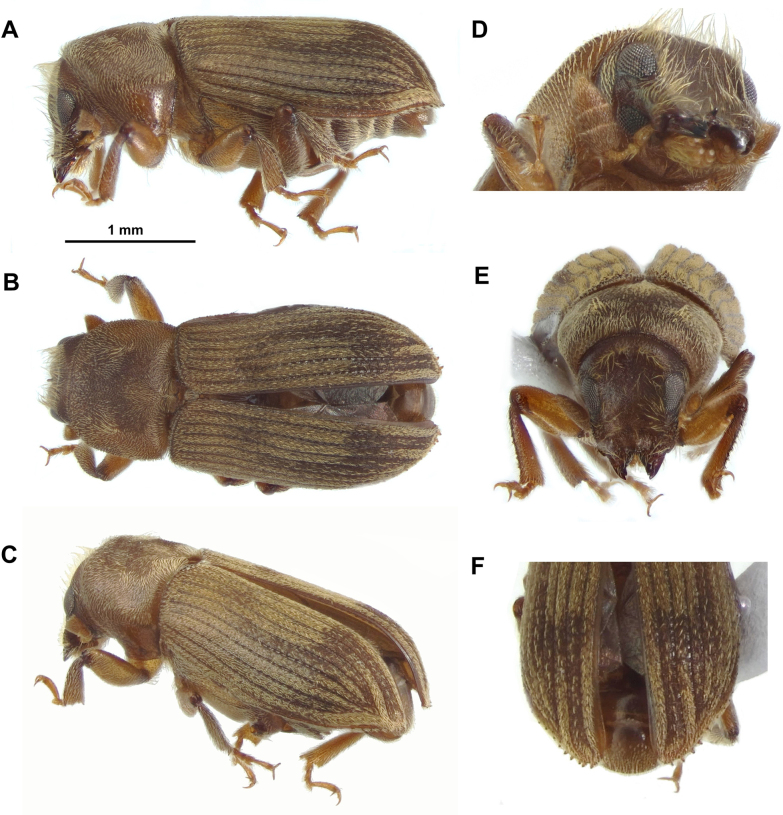
*Hyorrhynchus takakumaensis* Setiawan, Hata & Smith, sp. nov. male holotype. **A**. Lateral view; **B**. Dorsal view; **C**. Posterolateral view; **D**. Antenna; **E**. Frons; **F**. Declivity.

##### Type material.

***Holotype***: Japan • ♂; Kagoshima Prefecture, Tarumizu, Takakuma Experimental Forest of Kagoshima University; 31°31'53.1"N, 130°45'36.0"E; 581 m a.s.l.; secondary broadleaf forest, flight interception trap; 21.xi.24; Y. Setiawan leg.; ELKU. ***Paratype***: Japan • 1 ♂, same data as for holotype.

##### Diagnosis.

2.82–3.32 mm long (mean = 3.07 mm; *N* = 2), 2.35–2.77× as long as wide (mean = 2.56×; *N* = 2). Body brown, covered with yellow vestiture. Elytral disc bearing dense yellow setae and vestiture on interstriae 1–4; yellow setae are arranged in a suture-like pattern from the declivity to the apex; declivity to the apex bearing dense yellow setae and vestiture on interstriae 1–3. Frons bearing dense, erect, long, yellow hair-like setae along the dorsal and lateral margins; surface moderately covered. This species is most similar to *H. unicornis*, from which it can be distinguished by its smaller size and an apical epistomal margin with a broad median tubercle that gradually tapers apically, apex blunt, appearing horn-like in shape with a broad base in lateral view.

##### Description.

**Male**. 2.82 mm long, 2.35× as long as wide. Body brown, oblong, covered with yellow vestiture. ***Head***: epistoma strongly excavated, from epistoma to anterodorsal margin of the eyes, excavation bordering the ocular margin; surface shagreened; lateral margin lined with dense, long yellow hair-like setae; apical epistomal margin with a broad median tubercle, gradually tapering apically, apex blunt, appearing horn-like shape with a broad base in lateral view. Frons strongly excavated; excavation borders the ocular margin; surface shagreened; bearing dense, erect, long, yellow hair-like setae along the dorsal and lateral margins; surface moderately covered. Eyes divided. Antennal club elongate, with two transverse sutures. ***Pronotum***: 0.68× as long as wide; wider than long in dorsal view; disc flat; surface evenly covered with yellow vestiture; base bearing slightly elongate, yellow hair-like setae; disc bearing slightly elongate, yellow hair-like setae concentrated on the right and left sides; pronotal slope without teeth. ***Scutellum***: small, linguiform; pubescent. ***Elytra***: 1.68× as long as wide; separately rounded at base from dorsal view; surface covered with yellow vestiture; disc bearing dense yellow setae and vestiture on interstriae 1–4; yellow setae are arranged in a suture-like pattern from the declivity to the apex; declivity to the apex bearing dense yellow setae and vestiture on interstriae 1–3; slightly elongate spines present along the apical margin of the elytra. ***Legs***: procoxae subcontiguous; uniformly brown.

**Female**. Unknown.

##### Etymology.

*Hyorrhynchus takakumaensis*: *takakuma*- = referring to Takakuma Experimental Forest of Kagoshima University, located in the Takakuma Mountains on the Osumi Peninsula, southern Kyushu, Japan; -*ensis* = Latin suffix meaning “originating from” or “pertaining to a place”. Refers to the locality where the species was collected. A variable adjective.

##### Distribution.

Japan, Kagoshima Prefecture, Tarumizu.

##### Biology.

Unknown.

##### Host plants.

Unknown.

### New records

#### Anisandrus auratipilus

Taxon classificationAnimaliaColeopteraCurculionidae

Smith, Beaver & Cognato, 2020

C9AFF76D-7179-52E7-80D8-0965C8C397B7

Fig. 2A–F

##### Material examined.

Japan • 3 ♀♀; Kagoshima Prefecture, Tarumizu, Takakuma Experimental Forest of Kagoshima University; 31°31'49.5"N, 130°45'09.2"E; 654 m a.s.l.; secondary broadleaf forest, flight interception trap; 05.iii.25; Y. Setiawan leg.; ELKU • 2 ♀♀; Kagoshima Prefecture, Tarumizu, Takakuma Experimental Forest of Kagoshima University; 31°32'26.2"N, 130°45'39.2"E; 627 m a.s.l.; secondary broadleaf forest, flight interception trap, 05.iii.25; Y. Setiawan leg.; ELKU.

**Figure 2.**
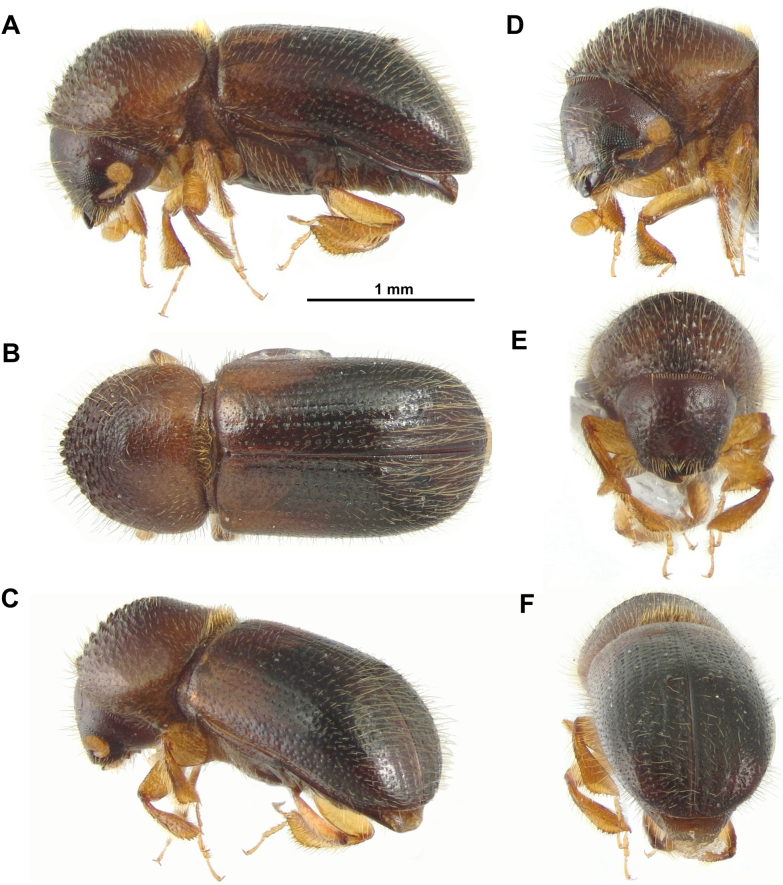
*Anisandrus auratipilus* Smith, Beaver & Cognato, 2020. **A**. Lateral view; **B**. Dorsal view; **C**. Posterolateral view; **D**. Antenna; **E**. Frons; **F**. Declivity.

##### Diagnosis.

2.52–2.76 mm long (*N* = 5); 2.38–2.46 times as long as wide. Body shiny, bicolor, pronotal and elytral bases brown, remaining parts dark brown. Legs and antennae light brown. Eyes shallowly emarginate, upper part smaller than lower part. Antennal club (type 1) (Hulcr et al. 2007). Pronotum from dorsal view round (type 0) (Hulcr et al. 2007); sides convex, conical anteriorly, from lateral view round (type 3) (Hulcr et al. 2007), short and tall, summit at midpoint; and the median basal region of the pronotum bears dense, erect yellow setae. Elytra have a small denticle on the declivital summit and minute denticle present on interstriae 2, three denticles also present on basal half interstriae 3; and the elytral declivity bears dense, erect yellow setae.

##### New records.

Japan (Kagoshima Prefecture, Tarumizu).

##### Distribution.

China (Fujian) (Smith et al. 2020).

##### Host plants.

Unknown.

##### Remarks.

This is only the second record of this species, which was originally described from Fuzhou, Fujian, China by Smith et al. (2020). This species is very similar to *Anisandrus apicalis* (Blandford, 1894), sharing a sharp, incurved spine or denticle at the base of the declivity on interstriae 2, but can be distinguished by its smaller size and bicolored appearance, as described in the diagnosis above.

#### Debus shoreae

Taxon classificationAnimaliaColeopteraCurculionidae

(Stebbing, 1907)

F3BF06A9-5608-5E99-872C-9D82CAB0D44B

Fig. 3A–F

Tomicus shoreae Stebbing, 1907: 39.
Debus shoreae (Stebbing): Beaver et al. 2014: 44.

##### Material examined.

Japan • 1♀; Kagoshima Prefecture, Tarumizu, Takakuma Experimental Forest of Kagoshima University; 31°31'30.4"N, 130°46'15.9"E; 523 m a.s.l.; secondary broadleaf forest, flight interception trap, 18.vi.25; Y. Setiawan leg.; ELKU • 1 ♀; Kagoshima Prefecture, Tarumizu, Takakuma Experimental Forest of Kagoshima University; 31°31'40.0"N, 130°46'09.4"E; 528 m a.s.l.; Japanese cedar forest, flight interception trap, 18.vi.25; Y. Setiawan leg.; ELKU.

**Figure 3.**
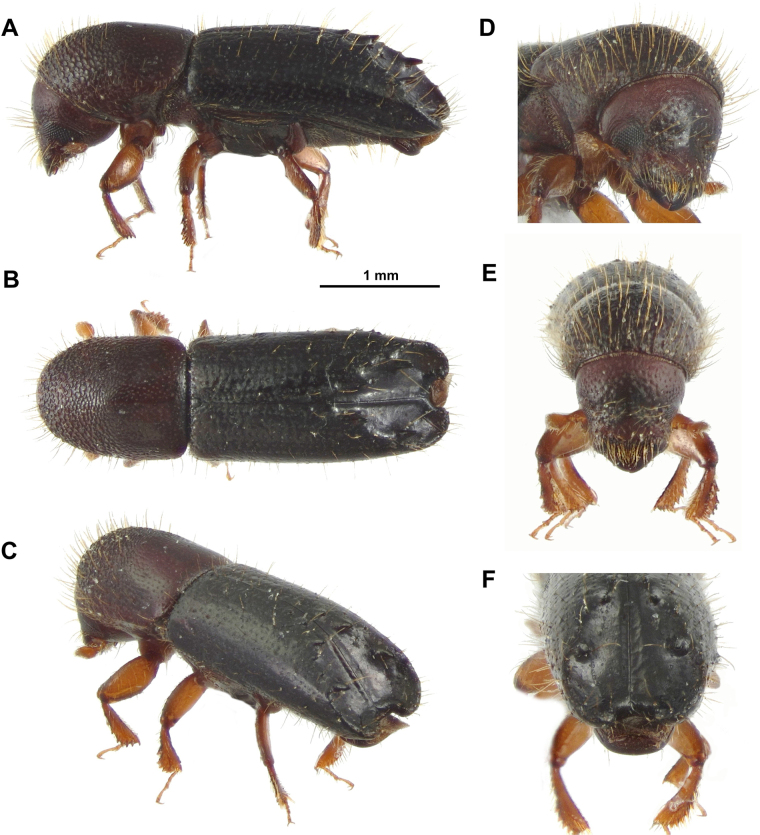
*Debus shoreae* (Stebbing, 1907). **A**. Lateral view; **B**. Dorsal view; **C**. Posterolateral view; **D**. Antenna; **E**. Frons; **F**. Declivity.

##### Diagnosis.

3.36–3.46 mm long (*N* = 2); 3.06–3.20 times as long as wide. Body uniformly dark brown, with the pronotum slightly lighter than the elytra. Legs and antennae brown. Pronotum elongate in dorsal view (type 9) (Hulcr et al. 2007). Elytra strongly excavated; declivity bears a small spine at its base and a longer spine at the middle of margin; declivity shallowly excavated, with a single row of strial punctures; and extensions of posterolateral elytral apex.

##### New records.

Japan (Kagoshima Prefecture, Tarumizu).

##### Distribution.

China (Guangxi, Sichuan), India (Arunachal Pradesh, Assam, Uttarakhand, Uttar Pradesh, West Bengal), Indonesia (Java, Sumatra), Laos, East Malaysia, Myanmar, Nepal, New Guinea, Thailand, Vietnam (Smith et al. 2020).

##### Host plants.

Polyphagous (Beaver et al. 2014; Smith et al. 2020).

##### Remarks.

This species is very similar to *Debus defensus* (Blandford, 1894), but can be distinguished by its larger size, and short but prominent posterolateral extensions at the apex of the elytra. This species is also similar to *Debus emarginatus* (Eichhoff, 1878) and is distinguished by the single row of strial punctures on the declivity.

## Discussion

*Hyorrhynchus* includes ten valid species, two of which were described from Japan. In Japan, only two *Hyorrhynchus* species have been recorded, *H. lewisi* and *H. unicornis*, both originally described from Hokkaido in northern Japan (Blandford 1894; Nobuchi 1966), where winters are colder and longer than in southern Japan. In our study, *H. takakumaensis* was collected in Kagoshima Prefecture, located in the southern part of Kyushu Island, in a secondary broadleaf forest at 581 m a.s.l., dominated by trees of Fagaceae and Lauraceae. However, the biology and host plants remain unknown because the specimens were collected using flight interception traps in autumn. The morphological features of *H. takakumaensis* are most similar to *H. unicornis* and can be distinguished by the apical epistomal margin with a broad median tubercle that gradually tapers apically, and the apex blunt, appearing horn-like in shape with a broad base in lateral view. *Hyorrhynchus takakumaensis* also differs from other *Hyorrhynchus* species in the pattern of setae and vestiture, the elytral disc bears dense yellow setae and vestiture on interstriae 1–4; the yellow setae are arranged in a suture-like pattern from the declivity to the apex; and the declivity to the apex bears dense yellow setae and vestiture on interstriae 1–3. *Hyorrhynchus takakumaensis* is a small species of *Hyorrhynchus*, at 2.82–3.32 mm long, and the proportion of this species body is 2.35–2.77× as long as wide.

In addition, we recorded two xyleborine ambrosia beetle species, *A. auratipilus* and *D. shoreae*, for the first time in Japan. There are currently three *Anisandrus* species in Japan: *A. apicalis*, *A. dispar* (Fabricius, 1792) and *A. maiche* (Kurentzov, 1941) (Smith et al. 2020). The genus is most diverse in montane forest habitats in Southeast Asia (Sittichaya et al. 2023). In Japan, only two species of *Debus* have been previously recorded, *D. defensus* and *D. emarginatus* (Park et al. 2020; Smith et al. 2020). *Debus* is common in tropical forests throughout South Asia to the Pacific islands (Smith et al. 2020). The discovery of these tropical to subtropical species may be related to the fact that the present collection site is located in a broadleaf evergreen forest region in southern Kyushu. Thus, further research in southern Japan may be promising for exploring the relationship between bark and ambrosia beetle fauna in warm-temperate and tropical-to-subtropical regions. With these findings, the diversity of the Japanese Scolytinae fauna increases to three *Hyorrhynchus* species, four *Anisandrus* species, and three *Debus* species.

### Key to Japanese species of *Hyorrhynchus* Blandford, 1894 (males only)

**Table:** 

1	Apical epistomal margin with a median tubercle	**2**
–	Apical epistomal margin without a median tubercle	***H. lewisi***
2	Small size, 2.82–3.32 mm long; apical epistomal margin with a broad median tubercle, gradually tapering apically in lateral view	***H. takakumaensis* sp. nov**.
–	Large size, 3.8 mm long; apical epistomal margin with a narrow median tubercle, steeply tapering apically in lateral view	***H. unicornis***

## Supplementary Material

XML Treatment for Hyorrhynchus

XML Treatment for Hyorrhynchus takakumaensis

XML Treatment for Anisandrus auratipilus

XML Treatment for Debus shoreae

## Additional information

### Artificial Intelligence (AI) use

No artificial intelligence tools were used in this study.

### Author ORCIDs

Y. Setiawan https://orcid.org/0000-0002-7346-1997

S.M. Smith https://orcid.org/0000-0002-5173-3736

Y. Sakamaki https://orcid.org/0000-0002-1759-1367

M. Tokuda https://orcid.org/0000-0001-7162-0715

H. Tarno https://orcid.org/0000-0003-0997-5843

K. Hata https://orcid.org/0000-0002-8029-4250

### Data availability

All of the data that support the findings of this study are available in the main text.
